# CGSDA: inferring snoRNA-disease associations via ChebNetII and GatedGCN

**DOI:** 10.3389/fgene.2025.1684484

**Published:** 2025-10-16

**Authors:** Yongfu Zou, Yusong Lu, Shanghui Lu, Zhanliang Wei, Le Li, Shuilin Liao, Ting Zeng, Yi Zhang, Rui Miao

**Affiliations:** ^1^ School of Mathematics and Physics, Hechi University, Hechi, China; ^2^ Department of Neurosurgery, The Second Nanning People’s Hospital, Nanning, China; ^3^ Basic Teaching Department, Zhuhai Campus of Zunyi Medical University, Zhuhai, China; ^4^ Faculty of Innovation Enginee, Macau University of Science and Technology, Taipa, China; ^5^ School of Tourism and Culture, Nanning Normal University, Nanning, China

**Keywords:** disease-snoRNA association, GNNS, ChebNetII convolutional network, gated graph convolutional network, multiplefeatures fusion, residual mechanism

## Abstract

**Introduction:**

Recent biomedical studies have highlighted the pivotal role of non-coding RNAs (ncRNAs) in gene regulatory networks, where they influence gene expression, cellular function, and the onset and progression of various diseases. Among these, small nucleolar RNAs (snoRNAs), a prominent class of small ncRNAs, have attracted considerable research attention over the past two decades. Initially recognized for their involvement in rRNA processing and modification, snoRNAs are now understood to contribute to broader biological processes, including the regulation of disease mechanisms, maintenance of cellular homeostasis, and development of targeted therapeutic strategies. With ongoing advancements, snoRNAs are increasingly regarded as promising candidates for novel therapeutic agents in cancer, neurodegenerative disorders, endocrine conditions, and cardiovascular diseases. Consequently, there is a growing demand for efficient, cost-effective, and environment-independent approaches to study snoRNAs, which has driven the adoption of computational methodologies in this domain.

**Methods:**

In this work, we propose a novel predictive framework, CGSDA, which integrates a ChebNetII convolutional network with a gated graph sequence neural network to identify potential snoRNA–disease associations. The model begins by constructing a snoRNA–disease association network, embedding residual mechanisms into both modules to effectively capture the representations of snoRNAs and diseases. These representations are then fused and dimensionally reduced, after which the refined embeddings are fed into a predictor to generate association predictions.

**Results:**

Experimental evaluation demonstrates that CGSDA consistently outperforms baseline models in predictive accuracy. Ablation experiments were conducted to assess the contribution of each module, confirming that all components substantially enhance overall performance and validating the robustness of the proposed method. Furthermore, case studies on lung cancer and breast cancer showed that 10 out of the top 15 and 12 out of the top 15 predicted snoRNA-disease associations were validated by existing literature, respectively, confirming the model’s effectiveness in identifying potential novel snoRNA-disease associations.

**Discussion:**

The implementation of CGSDA, along with relevant datasets, is publicly available at: https://github.com/cuntjx/CGSDA. This public release enables the research community to further validate and apply the framework, supporting advancements in computational identification of snoRNA–disease associations and facilitating progress in snoRNA-based therapeutic development, and ultimately benefiting human health.

## 1 Introduction

SnoRNAs are a class of ncRNAs predominantly located in the nucleolus of eukaryotic cells, where they play critical roles in RNA modification. They are generally categorized into two major groups: H/ACA box snoRNAs and C/D box snoRNAs. H/ACA box snoRNAs are primarily responsible for guiding pseudouridylation, whereas C/D box snoRNAs direct site-specific methylation. Accumulating evidence indicates that methylation and pseudouridylation, both mediated by snoRNAs, are essential processing steps in the maturation of precursor rRNA into functional rRNA. Notably, a single snoRNA molecule typically serves as a guide for no more than two individual RNA modification sites. Beyond these canonical functions, some snoRNAs also exhibit non-traditional roles; for example (Ender et al., 2008; Babiarz et al., 2008; Taft et al., 2009), and HBII-180C (Ono et al., 2010) can function as microRNAs (miRNAs).

### 1.1 SnoRNA involmente in disease

Emerging evidence demonstrates that snoRNAs are differentially expressed and participate in key biological processes such as apoptosis, proliferation, and differentiation. Owing to their multidimensional regulatory functions, elucidating the molecular mechanisms through which snoRNAs influence disease development has become a major frontier in biomedical research over the past 2 decades. For instance, Tang et al. (2019) investigated the role of snoRNAs in non-small cell lung cancer (NSCLC) using both *in vitro* and *in vivo* loss-of-function analyses. Their study revealed that SNORA71A functions as an oncogene in NSCLC and contributes to disease progression. Similarly, Liu et al. (2022) examined the role of SNORD1C in colorectal cancer through multiple experimental approaches, demonstrating that this snoRNA is involved in several tumor-related processes and plays a critical role in cancer progression. Xu et al. (2014) employed quantitative RT-PCR to assess snoRNA expression in tissue samples from over one hundred hepatocellular carcinoma (HCC) patients, further validating their findings using cell-based experiments and a xenograft nude mouse model. Their results showed that upregulation of SNORD113-1 inhibited HCC growth, suggesting its potential utility as both a diagnostic biomarker and therapeutic target. Beyond these individual studies, a growing body of evidence underscores the involvement of snoRNAs in diverse human diseases. In 2022, Huang et al. (2022) published a systematic review summarizing the biological functions and mechanistic roles of snoRNAs in tumor pathophysiology. Subsequently, Chabronova et al. (2024) provided a comprehensive review focusing on snoRNAs in cardiovascular development, physiology, and heart-related disorders. Their work not only consolidated current findings but also highlighted the potential clinical applications of snoRNAs in cardiovascular medicine. In the same year, Shen et al. (2024) conducted another systematic review that emphasized the diagnostic and therapeutic significance of snoRNAs across multiple disease contexts.

In addition to studies on human diseases, several investigations have explored the association between snoRNAs and animal pathologies (Omonijo et al., 2023; Regårdh et al., 2022; Anderson et al., 2022). Such cross-species comparative studies not only confirm the broad involvement of snoRNAs in disease processes across species but also help elucidate the conserved functional mechanisms of snoRNAs in disease development. Therefore, exploring the association between snoRNAs and diseases can help reveal the complex mechanisms of disease occurrence and development and, ultimately, benefit clinical applications.

### 1.2 Computational approaches for snoRNA–disease association prediction

As a methodological system characterized by high efficiency in processing large-scale data, strong scalability, and cross-domain adaptability, computational approaches have become deeply embedded in multidisciplinary and interdisciplinary research fields such as bioinformatics, drug discovery, and systems biology. These methods, supported by the rapid improvement in the cost–performance ratio of computational resources and the iterative advancement of deep learning architectures, now represent a central paradigm for the integration and analysis of multimodal data. In the context of snoRNA–disease association prediction, computational methods have been extensively applied, offering advantages of low cost, high timeliness, and broad generalizability when used as guidance tools. To the best of our knowledge, the first computational framework developed for this purpose was the iSnoDi-LSGT model, proposed by Zhang and Liu (2022). This model integrates dual constraints with topological node embeddings to predict potential snoRNA–disease associations. Building upon this foundation, several computational frameworks have since emerged. For example, Sun et al. (2022) introduced the PSnoD model, which draws inspiration from matrix completion techniques. Their framework constructs three networks and incorporates bounded nuclear norm regularization into a matrix completion strategy to enhance prediction Acc. Momanyi et al. (2024) proposed SAGESDA, a graph neural network–based approach that fuses multiple networks to build snoRNA–disease heterogeneous networks. They then applied the GraphSAGE algorithm to learn node representations, followed by a dot product classifier for association inference.

Zhang et al. further advanced the field by developing GCLSDA, a method based on a lightweight graph convolutional network (GCN). This approach first extracts node representations of snoRNAs and diseases via a light GCN (LightGCN), and then applies a contrastive learning mechanism to mitigate the challenges of sparse correlation matrices and node embedding over-smoothing, thereby improving model performance (Zhang et al., 2023). Similar GCN-based or variant methods include GCNSDA (Liu et al., 2021), GCASDA (Liu et al., 2024) and IGCNSDA (Hu et al., 2024). Beyond GCNs, Muna et al. (2025) proposed GBDTSVM, a hybrid framework combining two classical machine learning algorithms: gradient-boosted decision trees (GBDT) and support vector machines (SVM). In this method, GBDT is used to extract node features, which are subsequently passed to the SVM classifier for snoRNA–disease prediction. More recently, La Rosa et al. proposed GL4SDA, a novel framework that integrates multiple modalities. This model derives snoRNA representations from their secondary structures, leverages large language models (LLMs) to generate disease features, and finally employs a graph neural network with an attention mechanism to predict snoRNA–disease associations. As shown in Table 1, we have provided a brief summary of the state-of-the-art methods.

**TABLE 1:** The brief summary of the state-of-the-art methods.

Name	Main approach	Strengths	Limitations
iSnoDi-LSGT	This method first obtains local similarity constraints between snoRNAs and diseases, as well as global topological constraints, then combines non-negative matrix factorization to predict potential snoRNA-disease associations	The local similarity constraints and global topological constraints proposed by the model enable efficient identification of potential snoRNA-disease associations. The authors have deployed the model on a web server, allowing users to utilize it directly without the need for training	It relies on low-rank assumptions and struggles to fully capture high-order embeddings of nodes
GCNSDA	This computational framework constructs association networks based on snoRNA-disease bipartite graphs, then employs GCN for prediction	It can effectively capture local topological structures and node feature correlations in graph data	It struggles to capture long-range dependencies in graphs due to over-smoothing and exhibits high computational complexity
IGCNSDA	This model is built on the GCN framework combined with a subgraph generation algorithm. It first employs a GCN module to learn embeddings of snoRNAs and diseases, then refines these embeddings through the subgraph generation algorithm. Finally, it predicts snoRNA–disease associations by computing the dot product between the final embeddings of snoRNAs and diseases	The proposed subgraph generation algorithm not only effectively enhances the model performance but also endows the model with a certain degree of interpretability	The model is plagued by the over-smoothing issue, which in turn reduces the model’s interpretability
PSnoD	This model employs multiple networks to construct heterogeneous snoRNA-disease networks, then utilizes bounded nuclear norm regularization (BNNR) to predict potential snoRNA-disease associations	Owing to the incorporation of additional constraints and regularization terms into the model, the model exhibits robust resistance to data noise	Reliance on low-rank assumptions, limited ability to fully capture higher-order embeddings of nodes
GCLSDA	This model integrates LightGCN with contrastive learning. LightGCN is first applied to learn representations of snoRNAs and diseases. Contrastive learning is then introduced to alleviate the negative effects of both the sparsity of the snoRNA–disease association matrix and the oversmoothing problem inherent in LightGCN. Similar to IGCNSDA, GCLSDA predicts potential snoRNA–disease associations by computing the dot product between the final embeddings of snoRNAs and diseases	The model alleviates the adverse impacts of sparse association matrices and over-smoothing on model performance by integrating contrastive learning. Additionally, it incorporates noise augmentation techniques to further enhance prediction accuracy	The integration of contrastive learning increases the instability of the model during training
SAGESDA	This model begins by integrating multiple networks to construct a snoRNA–disease heterogeneous network. Based on this heterogeneous structure, GraphSAGE is employed to learn feature representations of the nodes. Finally, potential snoRNA–disease associations are inferred using a dot product classifier applied to the learned embeddings	By adopting mini-batch gradient descent technology to partition the graph into smaller subgraphs, the model enhances its accuracy, training efficiency, and generalization ability	The over-smoothing of the GraphSAGE module exerts an adverse impact on the model performance
GL4SDA	This framework first leverages snoRNA secondary structures to generate representations of snoRNAs. For diseases, feature representations are derived using a LLMs. These embeddings are then integrated into a graph neural network with an attention mechanism, which is employed to predict potential snoRNA–disease associations	The model effectively enhances its performance by leveraging snoRNA secondary structure information and adopting large language models to generate disease embeddings	The over-smoothing issue in the model’s GNN module exerts an adverse impact on the model performance
GCASDA	This model is built upon the GCN framework and incorporates a multi-view graph attention mechanism. By integrating multiple perspectives of the snoRNA–disease network, GCASDA enhances feature learning and predicts potential snoRNA–disease associations	The model effectively enhances its performance by leveraging the global features and interaction features of snoRNA-disease node pairs	The efficiency of computing the global features and interaction features of snoRNA-disease node pairs decreases significantly as the data volume increases
GBDTSVM	This computational framework integrates two traditional machine learning methods, GBDT and SVM, for predicting potential snoRNA–disease associations. In this model, GBDT is first employed to extract node representations, which are then input into the SVM classifier to perform association prediction	By leveraging the strong feature extraction capability of GBDT and the classification advantages of SVM, the model achieves high-accuracy prediction with low computational cost	As a traditional machine learning model, it may have limitations in handling high-order feature interactions in complex graph-structured data compared to deep graph neural networks

Although the past 2 decades have yielded substantial evidence supporting snoRNA–disease associations, the systematic collection of relevant data has lagged behind. This limitation has hindered the effective application of computational methods in this field, as these approaches typically require large-scale datasets to achieve optimal performance. Consequently, the relatively limited availability of high-quality data has been one of the key factors restricting broader computational applications in snoRNA–disease association prediction. Furthermore, there are relatively few high-performance models in the field of snoRNA-disease association prediction, and most of them adopt a single graph neural network for feature extraction or association prediction, which may bring the following adverse impacts on model performance. First, the over-smoothing of graph neural networks can degrade model performance. Second, relying solely on one type of neural network for feature extraction may exert an adverse impact on model performance due to the learning bias of the neural network. To address this gap and to further advance the development and optimization of predictive models, we propose a novel framework, CGSDA, designed to identify potential snoRNA–disease associations. CGSDA integrates two GNN modules: the ChebNetII convolutional network (ChebNetII) and gated graph convolutional network (Gated). The framework operates in three main stages. First, a snoRNA–disease association network is constructed, with a residual mechanism embedded into both modules to alleviate over-smoothing during representation learning. Next, the node embeddings learned by the two graph neural network modules are fused and subjected to dimensionality reduction, aiming to mitigate the adverse impact of the learning bias of a single neural network on model performance. Finally, the reduced-dimensional representations are passed into an inner product decoder to generate predictions of potential snoRNA–disease associations. Comparative experiments demonstrate that CGSDA consistently outperforms baseline models in prediction Acc. Furthermore, ablation studies reveal that each component of the framework contributes significantly to overall performance, thereby confirming the effectiveness and robustness of the proposed model.

## 2 Materials and methods

### 2.1 Dataset

In this study, two datasets (i.e., dataset SDAD and dataset MNDR) were employed. Specifically, Dataset SDAD was curated by La Rosa et al. (2025) and served as a subset of the RNADisease v4.0 database. SDAD encompasses 60 diseases, 384 snoRNAs, and 911 experimentally validated snoRNA-disease associations. Dataset MNDR, by contrast, was collected by Sun et al. (2022), and it contains 27 diseases, 220 snoRNAs, and 459 experimentally validated snoRNA-disease associations. Detailed information about the SDAD dataset is available at https://github.com/BCB4PM/GL4SDA. Dataset MNDR can be downloaded from https://github.com/linDing-groups/PSnoD or https://github.com/mariamuna04/gbdtsvm. We place the basic information of the two datasets in Table 2. In this work, we represent the snoRNA–disease association network as a graph, where snoRNAs and diseases serve as the nodes. We use $N_{S}$ and $N_{D}$ to denote the number of snoRNAs and diseases, respectively, and thus we have $N_{S}=384$ and $N_{D}=60$ in SDAD dataset. Here, we use S to denote the set consisting of snoRNAs, denoted as $S=\left\{s_{1},\ldots ,s_{N_{s}}\right\}$ . Similarly, we use D to denote the set consisting of diseases, denoted as $D=\left\{d_{1},\ldots ,d_{N_{D}}\right\}$ . The complete set of nodes is then given by V={S,D} . We define the adjacency (association) matrix of the snoRNA–disease association network as A , where each element i th row and j th column is specified according to Equation 1:

$$A_{ij}=\left\{\begin{array}{cc} 1,\; & \begin{array}{c} \text{if snoRNA }s_{i}\left(1\leq i\leq N_{S}\right)\text{ is associated }\\ \text{ with disease }d_{j}\left(1\leq j\leq N_{D}\right) \end{array}\\ 0,\; & \text{unconfirmed or unknown} \end{array}\right.$$ (1)

**TABLE 2:** The basic information about SDAD and MNDR dataset.

	Name	Dataset: SDAD	Dataset: MNDR
		Number	Number
Min degree	snoRNAs	1	1
	Diseases	1	1
Max degree	snoRNAs	9	7
	Diseases	174	166
Average degree	snoRNAs	2.37	2.09
	Diseases	15.17	17
Median degree	snoRNAs	2	2
	Diseases	4	3
Total	snoRNAs	384	220
	Diseases	60	27
	Associations	911	459

During the training phase, all experimentally validated snoRNA–disease associations are treated as positive samples, while the remaining unobserved pairs are regarded as negative samples. To construct the training set, a subset of the positive samples is randomly removed from the original snoRNA–disease association matrix A . The resulting matrix, denoted as $A_{\mathrm{train}}$ , is then used for model training.

### 2.2 Initial features of snoRNA

There are numerous methods for extracting features from RNA molecules, and the choice of method can significantly influence downstream tasks. Among them, the k-mer algorithm is one of the most widely used approaches due to its efficiency and broad applicability. It is implemented in several feature extraction tools such as Jellyfish, KMC, and Kraken. The k-mer algorithm has two key capabilities: (1) it counts k-mer occurrence frequencies to capture sequence composition patterns, repetitive regions, and mutation hotspots; and (2) it can be adapted to different applications by varying the value of k. For instance, setting k=6 allows for the characterization of six-nucleotide conserved regions within RNA sequences, which can help identify potential miRNA binding sites. Recent studies have systematically evaluated the impact of different k values on the performance of various downstream tasks (e.g., Vinje et al. (2015); Chor et al. (2009); Tahara et al. (2023)). Despite its advantages, the k-mer algorithm faces challenges. Extremely long or short sequences may compromise feature quality, and conventional k-mer approaches typically focus only on linear sequence information, overlooking secondary structural features. However, accumulating evidence demonstrates that RNA secondary structure plays a critical role in determining function. For example, studies have confirmed that snoRNA activity is strongly influenced by its structural conformation (Ganot et al., 1997; Kiss-László et al., 1996). This highlights the necessity of employing feature extraction algorithms that incorporate structural information.

One such method is nRC, a tool specifically developed by Fiannaca et al. (2017) for non-coding RNA (ncRNA) feature extraction, differs from other methods that overlook ncRNA structural features in that it can integrate the secondary structure information of ncRNAs into feature representation, thereby improving feature quality. Previous studies (e.g., La Rosa et al. (2025)) have verified that nRC outperforms baseline methods in ncRNA-related prediction tasks, confirming its suitability for snoRNA feature extraction. Therefore, we directly used the features extracted by nRC as the initial features of snoRNAs in the CGSDA model.

### 2.3 Initial features of disease

There are multiple approaches to representing disease features. One of the earliest methods was one-hot encoding based on disease categories. However, while effective for simple classification, this representation has shown limited performance in more complex tasks. Another widely adopted approach is disease semantic similarity, introduced by Xuan et al. (2013), Schriml et al. (2012), which leverages the MeSH database (https://www.ncbi.nlm.nih.gov/) to compute ontology-based similarity between diseases (Lu et al., 2022; Ouyang et al., 2024). This method has since been applied extensively across several studies. Importantly, many diseases are described in detail through textual resources, including clinical manifestations, pathogenic mechanisms, diagnostic criteria, classification schemes, and progression patterns. These textual descriptions capture rich and evolving knowledge about diseases, yet traditional feature extraction methods often overlook this valuable source of information.

Large Language Models (LLMs) can effectively understand the contextual information of text and generate corresponding numerical features as needed. The bge-icl-en tool, developed by Li et al. (2024) from the Beijing Academy of Artificial Intelligence (BAAI), is capable of converting English textual information into word embeddings. This tool adopts an open-source model and can be downloaded and deployed by users via https://github.com/FlagOpen/FlagEmbedding. We chose to generate the initial features of diseases by inputting the textual summaries of diseases from the MalaCards database (Rappaport et al., 2013) into the bge-icl-en tool, based on the two key considerations.

First, the MalaCards database (https://www.malacards.org/) integrates dozens of data sources and provides detailed textual descriptions for more than 16,000 human diseases. The textual information about the disease in MalaCards, including the status and classification of the disease, pathogenesis, characteristics and impacts of the disease, etiology and risk factors, preventive interventions, disease management and prognosis, contains rich semantic details, which are crucial for distinguishing disease characteristics. Unlike traditional methods (e.g., one-hot encoding, ontology-based semantic similarity), LLMs (such as bge-icl-en) can capture contextualized semantic information, avoiding the limitations of disease feature representation.

Second, the bge-icl-en tool has demonstrated excellent performance in multiple text embedding benchmark tests (Li et al., 2024) and supports open-source deployment, which not only ensures the accuracy of disease feature extraction but also guarantees the reproducibility of experimental results.

Specifically, we used the “Summary” field in the disease entries of MalaCards as the input to bge-icl-en, and the generated embedding vectors were used as the initial features of diseases.

### 2.4 CGSDA

Spectral convolutional networks represent an important class of graph neural networks with broad applications across diverse tasks. Among the most representative models are ChebNet (Defferrard et al., 2016) and GCN (Kipf and Welling, 2016), both of which perform spectral graph convolutions using Chebyshev polynomials. Notably, GCN can be viewed as a simplified variant of ChebNet, as it relies only on the first two Chebyshev polynomials. Despite this simplification, GCN often outperforms ChebNet in practice. To address this limitation, the ChebNetII model was introduced, which enhances Chebyshev polynomial approximation through Chebyshev interpolation, thereby mitigating the Runge phenomenon and improving model performance. Given its demonstrated superiority over baseline models, we adopted ChebNetII in this study to extract feature representations of snoRNAs and diseases.

**FIGURE 1:**
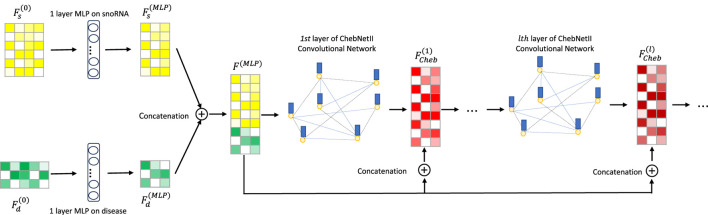
The overview of CGSDA framework. Embedding extracted by ChebNetII.

Li et al. (2015) integrated gated loop units, optimization techniques, and graph neural networks to propose GGNNs, which are more effective than traditional sequence models in extracting node embeddings from graphs. As is well known, deep learning models exhibit “preference learning” toward certain samples. To enhance both performance and robustness, we combine the ChebNetII model with the Gated model, introducing the CGSDA framework for predicting potential snoRNA–disease associations. The overall structure of the CGSDA model is illustrated in Figures 1, 3. At a high level, the core process of the model can be divided into three main steps.

Step 1 The initial features of snoRNAs and diseases are first passed through a single-layer multilayer perceptron (MLP) for dimensionality reduction. The resulting downscaled features are then concatenated and input into the multilayer ChebNetII network. To mitigate the negative effects of the “oversmoothing” problem commonly observed in graph neural networks, we introduce a residual mechanism that concatenates the downscaled representations with the inputs of each ChebNetII layer. Finally, the output of the terminal ChebNetII layer is fed into another single-layer MLP for further refinement and dimensionality reduction of the node embeddings, yielding the final output $F_{\mathrm{Cheb}}^{\left(L\right)}$ of the ChebNetII module. The detailed process is illustrated in Figure 1.

**FIGURE 2:**
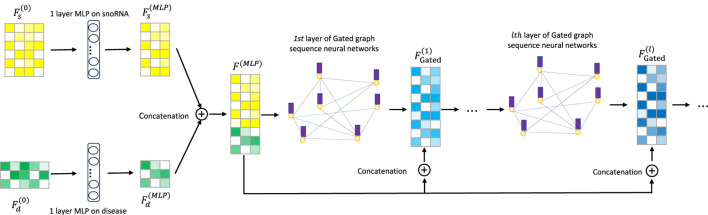
The overview of CGSDA framework. Embedding extracted by gated graph sequence neural networks.

Step 2 In this step, the ChebNetII module is replaced with the Gated module, while following a similar process to extract snoRNA and disease features. Specifically, the initial features of snoRNAs and diseases are first reduced in dimension using a single-layer MLP. To address the oversmoothing issue in GNNs and its adverse effect on model performance, the reduced features are then concatenated into the multilayer GGNN, enhanced with a residual mechanism. After processing, the Gated module produces the final output $F_{\mathrm{Gated}}^{\left(M\right)}$ . The detailed workflow is illustrated in Figure 2.

**FIGURE 3:**
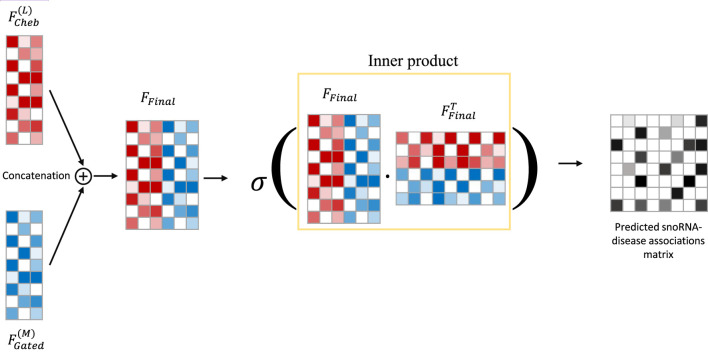
The overview of CGSDA framework. Embedding fusions and predicting potential snoRNA-disease associations.

Step 3 In the final step, the embeddings $F_{\mathrm{Cheb}}^{\left(L\right)}$ and $F_{\mathrm{Gated}}^{\left(M\right)}$ , obtained from Steps 1 and 2, are concatenated to form the final node embedding $F_{\mathrm{Final}}$ . An inner product is then computed between $F_{\mathrm{Final}}$ and its transpose $F_{\mathrm{Final}}^{T}$ to predict potential snoRNA–disease associations. This process is formalized in Equations 14, 15, and the detailed workflow is illustrated in Figure 3.

#### 2.4.1 Embedding extracted by ChebNetII

To accommodate both the ChebNetII and Gated modules, we define the association matrix and the initial feature representations using Equations 2, 3. Specifically, $F_{s}^{\left(0\right)}$ denotes the initial features of snoRNAs extracted with the nRC tool, while $F_{d}^{\left(0\right)}$ represents the initial features of diseases generated by the bge-icl-en tool. Furthermore, MLP refers to a single-layer MLP network.

$$Y=\left[\begin{array}{cc} 0 & A_{\mathrm{train}}\\ A_{\mathrm{train}}^{T} & 0 \end{array}\right],$$ (2)

$$F^{\left(MLP\right)}=\left[\begin{array}{c} F_{s}^{\left(MLP\right)}\\ F_{d}^{\left(MLP\right)} \end{array}\right],F_{s}^{\left(MLP\right)}=MLP\left(F_{s}^{\left(0\right)}\right),F_{d}^{\left(MLP\right)}=MLP\left(F_{d}^{\left(0\right)}\right).$$ (3)

For the ChebNetII module, the process of learning node embeddings can be formally expressed as Equations 4:

$$F_{\mathrm{Cheb}}^{\left(m+1\right)}=\frac{2}{K+1}\overset{K}{\underset{k=0}{\sum }}\overset{K}{\underset{j=0}{\sum }}\gamma_{j}T_{k}\left(x_{j}\right)T_{k}\left(\hat{L}\right)F_{\mathrm{Cheb}}^{\left(m\right)},\;m=0,\ldots ,M.$$ (4)

In this equation, M denotes the number of network layers in the ChebNetII module, and $F_{\mathrm{Cheb}}^{\left(0\right)}=F^{\left(MLP\right)}$ . The parameter K represents the order of the Chebyshev polynomial, where $T_{k}\left(x_{j}\right)=cos\left(k\cdot arccos\left(x_{j}\right)\right)$ defines the Chebyshev polynomial and $x_{j}=cos\left(\frac{\left(j+0.5\right)\pi }{K+1}\right)$ . The polynomial order K is a critical parameter linking the theoretical foundation of Chebyshev polynomials to their practical applications. Both theory and empirical studies have shown that lower-order polynomials are effective for approximating smooth and gradually varying functions, whereas higher-order polynomials are required to capture more complex functional relationships. However, although increasing the order improves the model’s capacity to fit complex functions and enhances approximation Acc., it also leads to higher computational costs and potential overfitting. Therefore, selecting an appropriate order requires balancing Acc., efficiency, and stability according to the specific task. Notably, ChebNetII becomes approximately equivalent to GCN when K=1 . Furthermore, $\hat{L}$ represents the scaled Laplacian matrix of the adjacency matrix Y and $\gamma_{j}$ are the learnable parameters for j=0,1,…,K .

#### 2.4.2 Embedding extracted by GatedGCN

Similar to the ChebNetII module, the GatedGCN module begins by feeding the initial features of snoRNAs and diseases into a single-layer MLP for learning and dimensionality reduction. The resulting feature representations are then concatenated and reintroduced into the GatedGCN network to generate node embeddings. The learning process of node representations within the convolutional layers of the GatedGCN network can be formally described by Equations 5–7:

$$h_{v}^{\left(0\right)}=F_{v}^{\left(MLP\right)}\|0,v\in V.$$ (5)

$$\alpha_{v}^{\left(l+1\right)}=\underset{u\in N\left(v\right)}{\sum }e_{u,v}\cdot \Theta^{\left(l\right)}\cdot h_{v}^{\left(l\right)},\;l=0,\ldots ,L.$$ (6)

$$h_{v}^{\left(l+1\right)}=GRU\left(\alpha_{v}^{\left(l+1\right)},h_{v}^{\left(l\right)}\right).$$ (7)

Where L denotes the sequence length of the convolutional layer in the GatedGCN module. $h_{v}^{\left(l\right)},\;l=0,\ldots ,L$ represents the embedding vector of node v at the l -th convolutional layer, while $F_{v}^{\left(MLP\right)}$ is the representation of the node’s initial features after passing through a single-layer MLP. The symbol ‖ denotes a concatenation operation. N(v) refers to the set of nodes adjacent to node v , and $e_{u,v}$ indicates the edge weight from source node u to target node v , with a default value of 1. $\Theta^{\left(l\right)}$ denotes the learnable parameter matrix, while the gated recurrent unit (GRU) regulates the update of node embeddings. The operations of the GRU can be expressed as Equations 8–11.

$$\beta_{v}^{\left(l+1\right)}=\sigma \left(W_{\alpha \beta }^{\left(l+1\right)}\alpha_{v}^{\left(l+1\right)}+b_{\alpha \beta }^{\left(l+1\right)}+W_{\beta }^{\left(l+1\right)}h_{v}^{\left(l\right)}+b_{\beta }^{\left(l+1\right)}\right),\;l=0,\ldots ,L,$$ (8)

$$\zeta_{v}^{\left(l+1\right)}=\sigma \left(W_{\alpha \zeta }^{\left(l+1\right)}\alpha_{v}^{\left(l+1\right)}+b_{\alpha \zeta }^{\left(l+1\right)}+W_{\zeta }^{\left(l+1\right)}h_{v}^{\left(l\right)}+b_{\zeta }^{\left(l+1\right)}\right),$$ (9)

$$\eta_{v}^{\left(l+1\right)}=tanh\left(W_{\alpha \eta }^{\left(l+1\right)}\alpha_{v}^{\left(l+1\right)}+b_{\alpha \eta }^{\left(l+1\right)}+\beta_{v}^{\left(l+1\right)}⊙\left(W_{\eta }^{\left(l+1\right)}h_{v}^{\left(l\right)}+b_{\eta }^{\left(l+1\right)}\right)\right),$$ (10)

$$h_{v}^{\left(l+1\right)}=\left(1-\zeta_{v}^{\left(l+1\right)}\right)⊙\eta_{v}^{\left(l+1\right)}+\zeta_{v}^{\left(l+1\right)}⊙h_{v}^{\left(l\right)}.$$ (11)

Where σ is the sigmoid function, $W_{\alpha \beta }$ , $W_{\beta }$ , $W_{\alpha \zeta }$ , $W_{\zeta }$ , $W_{\alpha \eta }$ , $W_{\eta }$ , $b_{\alpha \beta }$ , $b_{\beta }$ , $b_{\alpha \zeta }$ , $b_{\zeta }$ , $b_{\alpha \eta }$ , $b_{\eta }$ are learnable parameters, and ⊙ is the Hadamard product.

#### 2.4.3 Residual mechanism and feature fusion

To mitigate the negative impact of “oversmoothing” on GNN performance, we incorporate a residual mechanism into the CGSDA model. As illustrated in Figure 1, the output features of the single-layer MLP are directly connected to the inputs of each layer in both the ChebNetII and GatedGCN modules. This operation can be formally expressed by Equations 12, 13.

$$F_{\mathrm{Cheb}}^{\left(l+1\right)}=ChebNetII\left(Y,F_{\mathrm{Cheb}}^{\left(l\right)}\|F^{\left(MLP\right)}\right),$$ (12)

$$F_{\mathrm{Gated}}^{\left(l+1\right)}=GatedGCN\left(Y,F_{\mathrm{Gated}}^{\left(l\right)}\|F^{\left(MLP\right)}\right).$$ (13)

Deep learning models often exhibit a “preferential learning” bias toward certain samples, which can negatively affect overall performance. To address this issue and enhance both the performance and robustness of the model, we fuse the embeddings obtained from the ChebNetII and GatedGCN modules, as defined in Equation 14.

$$F_{\mathrm{FINAL}}=F_{\mathrm{Cheb}}^{\left(L\right)}\|F_{\mathrm{Gated}}^{\left(M\right)}.$$ (14)

Where $F_{\mathrm{Cheb}}^{\left(L\right)}$ and $F_{\mathrm{Gated}}^{\left(M\right)}$ denote the outputs of the last layer of the ChebNetII and GatedGCN networks, respectively.

#### 2.4.4 Inner product decoder

After obtaining the final fused embeddings via Equation 14, we employed an inner product decoder to compute the predicted score for potential snoRNA-disease associations–this score quantifies the likelihood of a functional association between a given snoRNA and disease, serving as the core output of the CGSDA model for both training and inference. The calculation of the predicted score can be formalized in Equation 15:

$$\hat{Y}=\sigma \left(F_{\mathrm{FINAL}}\times {\left(F_{\mathrm{FINAL}}\right)}^{T}\right).$$ (15)

where $\hat{Y}$ denotes the predicted association scores between snoRNAs and diseases. The operation $F_{\mathrm{FINAL}}\times {\left(F_{\mathrm{FINAL}}\right)}^{T}$ computes the inner product between each pair of node embeddings–a higher inner product value indicates a stronger association likelihood. σ(⋅) denotes the sigmoid activation function, defined as $\sigma \left(x\right)=\frac{1}{1+e^{-x}}$ . Its role is to normalize the inner product results to the range (0,1) , converting the raw inner product values into probability-like predicted scores: a score close to one indicates a high likelihood of a snoRNA-disease association, while a score close to 0 indicates a low likelihood.

During model optimization, we used the predicted scores $\hat{Y}$ and the ground-truth association matrix $A_{\mathrm{train}}$ to compute the binary cross-entropy loss. This loss quantifies the discrepancy between the predicted scores and the true association labels (1 for confirmed associations, 0 for unconfirmed ones), guiding the model to adjust parameters (e.g., weights in ChebNetII and GatedGCN modules) to minimize prediction errors.

### 2.5 Evaluation measures

Given that our work involves binary prediction in the field of snoRNA-disease association prediction and considers evaluating model performance across multiple dimensions, we employed seven widely used performance metrics. These are AUC, AUPR, F1 score (F1), Acc., Recall (Rec.), Specificity (Spe.), and Precision (Pre.). These values range between [0,1], with values closer to one indicating superior model performance. Among them, AUC quantifies a model’s ability to distinguish positive samples from negative samples; a value closer to one signifies better discrimination capability. AUPR serves as a complementary metric to AUC, typically reflecting the model’s balance between “prediction reliability” and “association capture completeness”. A value closer to one indicates superior overall performance in both aspects. The F1 score is the harmonic mean of precision and recall, aiming to balance these two metrics. A high F1 score indicates the model avoids both excessive false positives and false negatives. Precision is the proportion of correctly classified samples out of all samples, reflecting the overall prediction accuracy of the model. Recall measures the model’s ability to capture all true positive associations; high recall ensures the model does not overlook potential snoRNA-disease associations. Specificity quantifies the model’s ability to correctly identify non-associations. Complementary to recall, it ensures the model does not mislabel unconfirmed pairs as associations, thereby reducing unnecessary experimental validation burdens. Precision measures the proportion of predicted positive associations that are actually true. High precision indicates that the model’s top-ranked predictions possess high reliability.

## 3 Results

### 3.1 Parameters tuning and performance evaluation

CGSDA was implemented using PyTorch and PyG, and all experiments were conducted on an NVIDIA GeForce GTX 4060 GPU. To evaluate its predictive performance, we applied 10-fold cross-validation (10CV). Consistent with other deep learning–based association prediction methods, all experimentally verified associations were treated as positive samples, while unverified associations were regarded as negative samples during training. To mitigate the adverse effects of sample imbalance on model performance, we ensured that the number of positive and negative samples remained equal throughout the training phase.

The results of the 10-fold Cross-Validations (10CV) are detailed in Table 3. As presented in Table 3, the proposed computational method achieved an average AUC of 98.22 % , AUPR of 97.19 % , F1 score of 95.68 % , accuracy of 95.66 % , recall of 96.10 % , specificity of 95.22 % , and precision of 95.32 % with standard deviations of 0.70 % , 1.55 % , 0.76 % , 0.78 % , 1.56 % , 2.13 % , and 2.03 % respectively. On the MNDR dataset, the method yielded an average AUC of 98.31 % , AUPR of 97.21 % , F1 score of 95.58 % , accuracy of 95.54 % , recall of 96.63 % , specificity of 94.44 % , and precision of 94.60 % , with respective standard deviations of 1.04 % , 2.67 % , 1.06 % , 1.08 % , 1.65 % , 1.96 % , and 1.79 % .

**TABLE 3:** Ten-fold cross-validation results performed by CGSDA based on SDAD and MNDR.

Dataset:	SDAD						
Fold	AUC (%)	AUPR (%)	F1 (%)	Acc. (%)	Rec. (%)	Spe. (%)	Pre. (%)
1	98.17	98.13	94.82	94.78	95.60	93.96	94.05
2	97.85	94.98	96.46	96.43	97.25	95.60	95.68
3	98.64	98.13	95.72	95.60	98.35	92.86	93.23
4	98.46	98.01	95.91	95.88	96.70	95.05	95.14
5	98.34	96.71	95.37	95.33	96.15	94.51	94.59
6	97.97	96.50	95.37	95.33	96.15	94.51	94.59
7	98.50	98.76	95.45	95.60	92.31	98.90	98.82
8	96.53	94.27	94.59	94.51	96.15	92.86	93.09
9	98.53	98.77	95.91	95.88	96.70	95.05	95.14
10	99.21	97.68	97.21	97.25	95.60	98.90	98.86
Average	98.22	97.19	95.68	95.66	96.10	95.22	95.32
SD	0.70	1.55	0.76	0.78	1.56	2.13	2.03

Dataset:	MNDR						
Fold	AUC (%)	AUPR (%)	F1 (%)	Acc. (%)	Rec. (%)	Spe. (%)	Pre. (%)
1	99.73	99.47	97.85	97.83	98.91	96.74	96.81
2	98.70	98.94	96.17	96.20	95.65	96.74	96.70
3	97.05	97.32	95.03	95.11	93.48	96.74	96.63
4	98.74	98.47	95.14	95.11	95.65	94.57	94.62
5	98.75	98.64	94.62	94.57	95.65	93.48	93.62
6	97.25	93.43	94.62	94.57	95.65	93.48	93.62
7	98.66	98.16	95.24	95.11	97.83	92.39	92.78
8	99.15	98.91	95.74	95.65	97.83	93.48	93.75
9	96.41	91.34	94.68	94.51	97.80	91.21	91.75
10	98.65	97.43	96.74	96.70	97.80	95.60	95.70
Average	98.31	97.21	95.58	95.54	96.63	94.44	94.60
SD	1.04	2.67	1.06	1.08	1.65	1.96	1.79

### 3.2 Comparative performance with other latest methods based on 10CV

To further evaluate the performance of CGSDA, we conducted comparative experiments. In these experiments, we selected comparative models based on three criteria: (1) methodological diversity (covering matrix processing methods, GNNs, traditional machine learning, and large language model-integrated approaches) to validate the advantages of CGSDA across different paradigms; (2) recency (all published between 2021 and 2025) to ensure alignment with current research progress; and (3) relevance (explicitly designed for snoRNA-disease association prediction rather than general non-coding RNA-disease tasks). Based on these criteria, we identified nine baseline models, with a brief description of these models provided in Table 1. This comparison was conducted using 10-fold cross-validation on both the SDAD and MNDR datasets. The performance metrics for each model are presented in Table 4. It can be observed that our CGSDA model achieved the highest performance metrics on both datasets. Specifically, on the SDAD dataset, the AUC and AUPR values reached 98.22 % and 97.19 % , respectively, outperforming the second-best model GBDTSVM by 2.16 % . On the MNDR dataset, the AUC and AUPR values were 98.31 % and 97.21 % , respectively, also surpassing the second-best model GBDTSVM. This fully demonstrates the superior predictive capability of CGSDA compared to the nine baseline models. We attribute this advantage to three main factors: (1) By integrating two GNN architectures, CGSDA effectively alleviates the adverse effects of “preferential learning” biases in deep learning models. (2) The incorporation of a residual mechanism mitigates the oversmoothing problem in GNNs, preserving critical feature information and enhancing performance. (3) Adjusting the K -value in the ChebNetII module enables the model to capture complex node-level information more comprehensively.

**TABLE 4:** The comparison results of CGSDA model and other state-of-the-art models based on ten-fold cross-validation.

Dataset: SDAD							
Method	AUC (%)	AUPR (%)	F1 (%)	Acc. (%)	Rec. (%)	Spe. (%)	Pre. (%)
iSnoDi-LSGT	75.69	76.32	66.74	61.14	74.03	60.75	57.37
GCNSDA	91.48	93.54	83.48	83.28	84.61	82.04	82.18
IGCNSDA	76.81	76.68	65.49	60.06	75.86	61.54	57.55
PSnoD	90.04	89.29	88.40	88.59	86.96	90.22	89.89
GCLSDA	83.59	82.56	70.60	65.81	86.84	73.08	70.58
SAGESDA	77.41	76.97	70.14	66.81	77.73	79.33	63.81
GL4SDA	90.38	84.34	83.31	84.64	75.84	90.77	92.19
GCASDA	94.32	93.26	91.48	93.55	91.18	90.92	89.72
GBDTSVM	96.06	95.21	93.95	92.96	94.41	92.31	91.72
CGSDA (our)	98.22	97.19	95.68	95.66	96.10	95.22	95.32

Dataset: MNDR							
Method	AUC (%)	AUPR (%)	F1 (%)	Acc. (%)	Rec. (%)	Spe. (%)	Pre. (%)
iSnoDi-LSGT	78.56	74.29	76.78	73.37	88.04	58.70	68.07
GCNSDA	91.35	85.00	90.13	90.09	89.99	89.95	90.07
IGCNSDA	76.41	76.71	77.30	79.89	68.48	71.29	78.73
PSnoD	90.47	88.18	89.50	89.67	88.04	91.30	91.01
GCLSDA	84.58	82.98	81.34	78.57	93.41	63.74	72.03
SAGESDA	77.65	82.62	75.25	72.83	82.61	63.04	69.09
GL4SDA	90.50	89.68	90.50	90.76	88.04	93.48	93.10
GCASDA	94.80	91.71	91.89	91.85	92.39	91.30	91.40
GBDTSVM	96.10	96.43	92.15	92.85	93.65	88.04	88.89
CGSDA (our)	98.31	97.21	95.58	95.54	96.63	94.44	94.60

### 3.3 Parameters tuning

The parameters of our model can be categorized into four groups: (1) ChebNetII module parameters, including the number of network layers and the order of Chebyshev polynomials. (2) GatedGCN module parameters, such as the number of network layers and the sequence length of GatedGraphConv. (3) Output dimensionality of the network layers. (4) Training parameters, including learning rate, weight decay, dropout rate, and the number of training epochs. In this section, we will conduct systematic parameter tuning based on the SDAD dataset to examine the impact of the aforementioned four parameter categories on model performance and determine the optimal parameter settings for our framework.

#### 3.3.1 Optimizable parameters in the ChebNetII module

**FIGURE 4:**
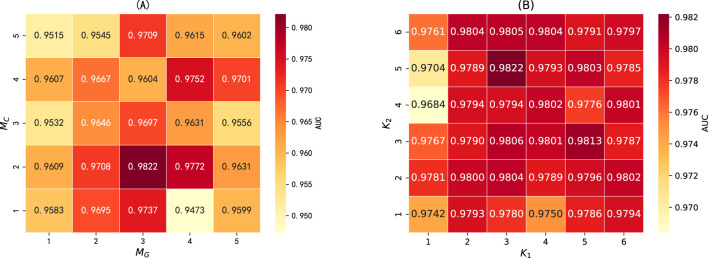
**(A)** The number of network layers in the ChebNetII and GatedGCN modules, where *M_C* and *M_G* represent the number of network layers in the ChebNetII module and GatedGCN module, respectively. **(B)** The order of Chebyshev polynomials, where *K_1* and *K_2* represent the order of the 1st layer and 2nd layer of the ChebNetII network, respectively.

• *The number of network layers for ChebNetII and GatedGCN modules.* Increasing the number of network layers in the ChebNetII and GatedGCN modules can enhance model complexity, thereby enabling the extraction of higher-order node features. However, an excessive number of layers may aggravate the “oversmoothing” problem and significantly increase the computational burden. To comprehensively evaluate the impact of network depth on overall model performance, we conducted joint tuning experiments on the number of layers in both modules. Specifically, the number of network layers for ChebNetII and GatedGCN was selected from the range {1,2,…,5} , denoted by $M_{C}$ and $M_{G}$ , respectively. As illustrated in Figure 4A, the model achieved optimal performance when the number of layers in the ChebNetII and GatedGCN modules was set to 2 and 3, respectively. Therefore, we adopted $M_{C}=2$ and $M_{G}=3$ for subsequent parameter tuning experiments.

• *The order of Chebyshev polynomials* K *.* Increasing the order of Chebyshev polynomials enables the model to capture more complex functions when learning node embeddings. However, excessively large orders can reduce computational efficiency and increase the risk of overfitting. In this study, the K values of the two-layer ChebNetII network were selected from the range 1–6. We denote the polynomial orders of the first and second layers in the ChebNetII module as $K_{1}$ and $K_{2}$ , respectively. As shown in Figure 4B, the model achieved the highest AUC when $K_{1}=3$ and $K_{2}=5$ .

#### 3.3.2 Optimizable parameters in the GatedGCN module

**FIGURE 5:**
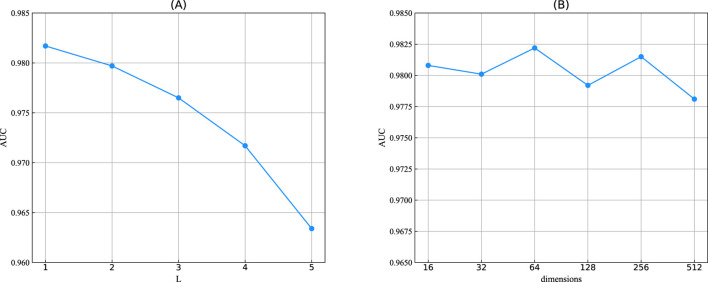
**(A)** The sequence length of the convolutional layer in the GatedGCN module, denoted by *L*. **(B)** The output dimensions of two modules.

• *The number of layers for GatedGCN module.* The tuning of network layers for the GatedGCN module was carried out jointly with that of the ChebNetII module, as described in Section 3.3.1).

• *The sequence length of the convolutional layer* L *.* We let L∈{1,2,…,5} . As can be seen in Figure 5A, the model performance improves as the value of L decreases and reaches an optimization at L=1 , after which it starts to decline. Therefore, in the CGSDA model we make L=1 .

#### 3.3.3 Optimization of network output dimensions and model training parameters

**FIGURE 6:**
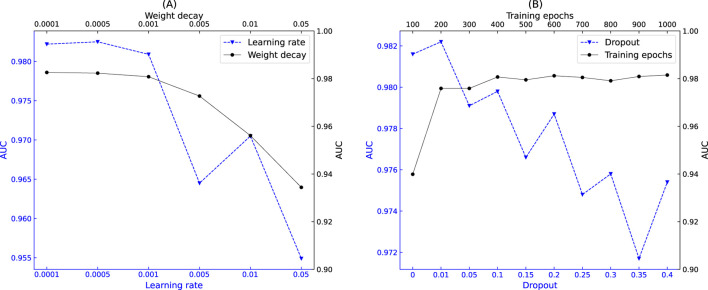
Impact of hyperparameters on model performance. **(A)** Relationship between learning rate, weight decay, and AUC. **(B)** Relationship between training epochs, dropout rate, and AUC.

• *The output dimensions of two modules.* To ensure proper concatenation of the embeddings generated by the ChebNetII and GatedGCN modules, we set their output dimensions to be equal. We evaluated model performance across six experiments with output dimensions {16,32,…,512} . As shown in Figure 5B, the model achieved its highest AUC when the output dimension was 64.

• *Learning rate and weight decay.* We applied the same learning rate and weight decay across all network layers of CGSDA. The learning rate was selected from { 0.0001,0.0005,…,0.1 } and the weight decay was chosen from {0.00005,0.0001,…,0.05} . As shown in Figure 6A, the model achieved its highest AUC when the learning rate and weight decay were set to 0.0005 and 0.0001, respectively.

• *Dropout and training epochs.* To ensure consistency during model training, we applied the same dropout rate across all network layers of CGSDA. The relationship between dropout and AUC is illustrated in Figure 6B. As shown, variations in dropout values caused fluctuations in model performance, with the highest AUC achieved at a dropout rate of 0.01. For training epochs, we conducted 10 experiments with epochs set to 100,200,…,1000 . From Figure 6B, we observe that AUC increased monotonically with training epochs up to 600, after which it declined. Therefore, we set the training epoch of CGSDA to 600.

### 3.4 Ablation tests

As noted earlier, our model primarily consists of two modules, ChebNetII and GatedGCN, with the residual mechanism incorporated into both. To evaluate the contribution of each component to overall performance, we designed three sets of ablation experiments based on the SDAD dataset. In the first group, CGSDA-C, the ChebNetII module was removed; in the second group, CGSDA-G, the GatedGCN module was removed; and in the third group, CGSDA-RES, the residual mechanism was removed.

We conducted ablation experiments using 10CV, and the results are summarized in Table 5. The findings show that removing individual components of the CGSDA model leads to a decline in overall performance. Specifically: (1) In comparison with CGSDA-C and CGSDA-G, the complete CGSDA model demonstrates that combining the two GNN modules effectively alleviates the negative impact of “preferential learning” on sample performance in deep learning models. (2) The inclusion of the residual mechanism significantly mitigates the oversmoothing problem in both modules, thereby enhancing overall model performance.

**TABLE 5:** Ablation tests.

Method	AUC (%)	AUPR (%)	F1 (%)	Acc. (%)	Rec. (%)	Spe. (%)	Pre. (%)
CGSDA-C	96.20	93.75	92.91	92.69	95.60	89.78	90.37
CGSDA-G	95.96	93.89	92.60	92.58	92.86	92.31	92.35
CGSDA-RES	95.24	94.21	91.75	92.06	89.01	95.05	94.72
CGSDA	98.22	97.19	95.68	95.66	96.10	95.22	95.32

## 4 Case study

To validate the predictive ability of our computational framework for novel snoRNA–disease associations in real-world applications and to assess its validity and reliability, we designed two case studies. The first evaluates the model’s predictive power for previously unseen diseases, while the second examines its ability to predict novel snoRNA–disease associations. The second case study follows a similar design to GL4SDA (La Rosa et al., 2025), GBDTSVM (Muna et al., 2025), and GCASDA (Liu et al., 2024), which predict new associations while retaining all existing snoRNA–disease pairs. In this work, we selected “lung cancer (DOID:1324)” and “breast cancer (DOID:1612)” for the first and second case studies, respectively. In the first case study, all snoRNAs associated with the target disease were removed before training, forcing the model to treat the disease as a new entity and predict its associations with all snoRNAs. In the second case study, only the unknown snoRNA–disease associations were treated as the test set. For both case studies, we ranked the prediction scores generated by the CGSDA model and selected the top 15 associations with the highest scores. These candidate associations were then validated through a literature search in the PubMed Biology database using the corresponding snoRNA–disease pairs as keywords. If an association was confirmed, it was annotated with the PMID of the supporting publication; otherwise, it was labeled as Unconfirmed. It should be noted that associations confirmed in the literature can only be used as evidence if they have been validated by experiments.

Lung cancer is a highly malignant disease characterized by poor treatment outcomes and high mortality worldwide. The prognosis depends heavily on the stage at diagnosis: patients with early-stage lung cancer have relatively high 5-year survival rates, whereas those diagnosed at advanced stages face a 5-year survival rate close to 0% . However, because early-stage lung cancer is typically asymptomatic, timely detection is challenging. Current screening methods rely primarily on medical imaging, which may result in missed diagnoses due to both limited visibility in early disease and dependence on the physician’s expertise. Consequently, identifying novel biomarkers for the early detection of lung cancer is of significant clinical importance. SnoRNAs have recently emerged as promising candidates in this regard. As shown in Table 6, 10 out of the top 15 snoRNAs predicted by the CGSDA model to be associated with lung cancer were validated by evidence from the literature.

**TABLE 6:** The top 15 predicted snoRNAs associated with lung cancer and breast cancer.

Lung cancer			Breast cancer		
Score	snoRNA	PMID	Ranking	snoRNA	PMID
0.9543	SNORD14C	29141226	0.9707	SNORD47	29793177
0.9171	SNORD112	32962511	0.9699	SNORDA55	36585466
0.9083	SNORD112-114	Unconfirmed	0.9518	SNORA25	Unconfirmed
0.8890	SNORA36B	Unconfirmed	0.9354	SNORA65	Unconfirmed
0.8787	SNORD116-4	Unconfirmed	0.9312	SNORA24	29287594
0.8552	SNORA12	25159866	0.9276	SNORD114-14	30647841
0.8415	SNORD113	32824183	0.9187	SNORD114-9	36585466
0.8201	SNORA71	31258730	0.9093	SNORD16	38311725
0.7911	SNORD29	32824183	0.9015	SNORA68	38594783
0.7594	SNORNAU50	32111002	0.8999	SNORD66	36585466
0.7276	SNORD114-6	Unconfirmed	0.8807	SNORD69	36585466
0.6803	SNORA47	31052265	0.8757	SNORD76	32160712
0.6719	SNOR38	32962511	0.8625	SNORD33	24260353
0.6400	SNORD96B	Unconfirmed	0.8581	SNORD49A	Unconfirmed
0.6377	SNORD3A	32962511	0.8244	SNORD74	32160712

Breast cancer is a common malignant tumor among women, though it also affects approximately 1 % of men (Jameson et al., 2020). While the etiology of breast cancer is not yet fully understood, it is well established that factors such as long-term improper diet, radiation exposure, heredity, and genetic mutations significantly increase risk. Epidemiological studies indicate that breast cancer remains the leading cause of cancer-related deaths among women in many countries, with lower mortality in economically developed regions likely due to early diagnosis and comprehensive treatment strategies (Sancho-Garnier and Colonna, 2019). In-depth research on breast cancer is therefore critical for elucidating its pathogenesis and risk factors, ultimately supporting early screening, effective treatment, and improved prognosis. As shown in Table 6, 12 of the top 15 snoRNAs predicted by our model to be associated with breast cancer have been validated in the literature.

## 5 Discussion and conclusion

snoRNAs, an important class of small ncRNAs, have attracted significant research attention over the past 2 decades. Studies of their functions have expanded from their initial roles in rRNA processing and modification to broader areas including disease mechanism regulation, cellular homeostasis, and targeted therapies. With ongoing research, snoRNAs are increasingly recognized as potential sources of novel therapeutic agents for cancer, neurodegenerative disorders, endocrine diseases, and cardiovascular conditions. Accordingly, there is an urgent need for efficient, cost-effective, and environmentally independent methods to study snoRNAs, which has driven the widespread adoption of computational approaches in this field. In this study, we developed CGSDA, a model that integrates the ChebNetII Convolutional Network (ChebII) module with the Gated GCN (Gated) module. The model first constructs a snoRNA–disease association network and incorporates a residual mechanism within both modules to learn the representations of snoRNAs and diseases. These node embeddings are subsequently fused and downscaled before being input into a predictor to infer potential snoRNA–disease associations. Experimental results demonstrate that CGSDA achieves superior predictive performance compared to baseline models. Furthermore, ablation experiments reveal that each component of the model makes a significant contribution to its overall performance, fully validating the effectiveness of the proposed framework.

In the comparative experiments, the CGSDA model exhibited excellent performance across multiple metrics with small standard deviations, demonstrating significant advantages over baseline models and confirming the good robustness of CGSDA. In the two types of case studies conducted, 10 and 12 out of the top 15 associations predicted by our model were validated by existing literature, respectively. This showcases the effectiveness of CGSDA in identifying biologically meaningful snoRNA-disease associations, indicating its great potential for guiding downstream experimental research.

Although CGSDA exhibits outstanding performance and practicality, it also has the following limitations: (1) The model is suitable for binary association prediction, and its predictive performance for multi-type associations requires further evaluation. (2) Although the nRC tool can integrate the secondary structure information of snoRNAs, it does not cover other key biological features of snoRNAs (such as expression levels, subcellular localization, and interactions with other RNAs), which may lead to the omission of important clues affecting association prediction. (3) Additional wet experiments are required to validate the predicted results. Based on this, in future work, we will expand the application of the CGSDA model in the field of multi-type association prediction and collaborate with medical schools to conduct wet experimental validation of the predicted results.

We attribute the superior performance of CGSDA to three main factors. First, by integrating the ChebNetII and GatedGCN modules, the model effectively mitigates the negative impact of “preference learning” inherent in deep learning models, as confirmed by our ablation experiments. Second, the incorporation of a residual mechanism helps reduce the “oversmoothing” phenomenon, further enhancing model performance. Third, adjusting the K value in the ChebNetII module allows the framework to capture complex node information more comprehensively, contributing to its predictive Acc.

## Data Availability

All the data and corresponding codes are available at https://github.com/cuntjx/CGSDA.
